# Experimental Investigation on Shear Behavior of the Interface between Early-Strength Self-Compacting Shrinkage-Compensating High-Performance Concrete and Ordinary Concrete Substrate

**DOI:** 10.3390/ma15144939

**Published:** 2022-07-15

**Authors:** Wenping Du, Caiqian Yang, Hans De Backer, Chen Li, Kai Ming, Honglei Zhang, Yong Pan

**Affiliations:** 1 Key Laboratory of Concrete and Prestressed Concrete Structures of the Ministry of Education, School of Civil Engineering, Southeast University, Nanjing 210096, China; ycqjxx@seu.edu.cn (C.Y.); 220201184@seu.edu.cn (K.M.); 2 Department of Civil Engineering, Ghent University, 9052 Zwijnaarde, Belgium; hans.debacker@ugent.be; 3 College of Civil Engineering and Mechanics, Xiangtan University, Xiangtan 411105, China; 230179447@seu.edu.cn; 4 Jiangsu Huatong Engineering Testing Co., Ltd., Nanjing 210000, China; dwpyls0802@gmail.com; 5 Jiangsu Eastern Expressway Management Co., Ltd., Lianyungang 222000, China; 201921002244@smail.xtu.edu.cn

**Keywords:** ESS-HPC, OCS, shear bond stress, surface roughness, bond stress–slip

## Abstract

To improve interface bonding stress, early-strength self-compacting shrinkage-compensating high-performance concrete (ESS-HPC) was selected as an excellent strengthening material to investigate by direct shear test. Tests on seventeen Z-type specimens were carried out considering the ESS-HPC and ordinary concrete substrate (OCS) compressive strength grade, the ESS-HPC curing age, the OCS surface roughness, and the ratio of steel shear dowels as the variables. A bond stress–slip model of the interface was proposed via statistical fitting. The results show that the surface roughness and ratios of steel shear dowels had the most important influence on the shear bond stress. The shear bond stress of the specimens without steel shear dowels increased by almost 15% as the ESS-HPC strength grade changed from C60 to C75. With the increase in the curing age, the shear bond stress showed a changing trend of first increasing and then decreasing. The coarser surface with the drilling method can improve the shear bond stress by 89%. To achieve a secondary increase in the shear bond stress of specimens with steel shear dowels, the minimum ratio of steel shear dowels was 0.83%. Analytical equations are proposed in combination with the CEB-FIB Model 2010 and AASHTO Model. The calculated results show reasonable agreement with the experimental results within an acceptable range.

## 1. Introduction

The core-filling strengthening method is often selected to strengthen and improve the shear behavior of hollow core bridges. However, the shear behavior between new and old concrete at the interface plays a significant role in this repair process. To solve this problem, the shear behavior of the interface has been investigated by many researchers. At present, the main focus has been on the influencing factors of the interface, including new concrete and ordinary concrete substrate (OCS) compressive strength, the OCS surface roughness, bonding agent types [1], curing age, the moisture state of the interface [2,3] and others. In the existing investigations, the new concrete has been mainly normal concrete, fiber-reinforced concrete [4,5,6], metakaolin geopolymer repair mortar [7], alkali-activated concrete [8], ultra-high fiber-reinforced concrete [9], self-compacting concrete [10], sand concrete [11], and recycled concrete [12]. The interface shear bond stress has mainly been investigated by slant [13], push-out [14,15,16,17,18,19], flexural, and splitting tests. However, there has been no unified expression of shear bond stress at the interface. Existing codes and experiments have been measured using different sizes, materials, and application conditions.

To improve the ductility behavior of the interface between new concrete and OCS, fibers have been used to improve the cohesion at the interface before the repair bond fully develops [20,21]. Simultaneously, many researchers have reported that the higher the compressive strength and coarser interface are, the higher interface shear stress is [22,23,24]. Zhang et al. [14,15] investigated the interface between carbonated concrete substrate and self-compacting concrete by direct shear tests. The results showed that the relationship between shear stress and carbonation depth is a quadratic function. The shear stress increased by 30% when the carbonation depth of the substrate was more than 20 mm. It was suggested that the strength difference between new concrete should not exceed 5 MPa. Therefore, the carbonated layer of the OCS surface should be conserved. Diab et al. [10] investigated slant shear bond stress between new self-compacting concrete and the OCS. The results showed that the stress can be improved by 26% when the OCS surface roughness was 6 mm in height and width. The shear behavior of the geometry of the prismatic specimen was more reliable than the cylindrical specimen. Mohamad et al. [25] mainly studied the friction and cohesion coefficients of the interface between new and old concrete under different normal stresses by push-off tests. The OCS surface was treated with five types of surface roughness (surface “left-as-cast”, deep groove, longitudinal roughened, transverse roughened, and indented). The average peak height was the best roughness parameter with which to evaluate the shear stress, and the empirical expression was proposed. According to three codes (ACI 318, Eurocode 2, and CEB-FIB Model 2010) and two authors (Santos and Gohnert), the calculated results obtained from the proposed equations showed good concordance with the experimental results within an acceptable range. Cattaneo et al. [26] studied the shear strength between new and old concrete under cycling loading by a typical push-off test and two other tests (beam-to-column and specific shape). The finite element analysis results showed that a specific shape with orthogonal restrains was more suitable to evaluate the cyclic behavior. The experimental evidence confirmed that the strength degradation occurred at the interface. Therefore, the static tests do not allow for evaluating the shear behavior. In the discussed theory, most investigations are based on the shear friction theory. However, the behavior of concrete crushing in the crack has not been considered in shear friction theory. Hwang et al. [27] proposed a softened strut-and-tie model to evaluate the interface shear strength. It can accurately predict the ultimate failure and pre-cracked shear planes. During the calculation process, the first cracking angle was equivalent to the inclination angle. However, this equivalent method was not suitable for high-strength concrete. The shrinkage of new and old concrete has a significant influence on interface stress. In the core-filling strengthening method, shrinkage-reducing admixture is added to new high-performance concrete to effectively reduce shrinkage [28]. Qin et al. [29] investigated the influence of shrinkage-reducing admixture on the interface between new and old concrete by four-point tests. The results showed that interfacial fracture toughness decreased with the extension of the duration of the moist environment condition. Meanwhile, shrinkage-reducing admixture can reduce the water diffusion coefficient and improve the interface integrity and durability. However, there is a lack of information on self-compacting concretes with excellent micro expansion, excellent ductility, and early strength. Therefore, our research team proposed a novel self-compacting concrete. i.e., early-strength self-compacting shrinkage-compensating high-performance concrete (ESS-HPC). At present, there have been few investigations into the interface behavior between ESS-HPC and OCS. Simultaneously, normal methods of shear bond stress considering the influence of the curing age are scarce.

The aim of this study is to address the gap in knowledge, and it was carried out by testing seventeen groups of Z-type specimens. Four different strength grades (C60, C65, C70, and C75), three curing ages (7 days, 28 days, and 56 days), five different surface roughnesses (R0, R1, R2, R3, and R4), and three ratios of steel shear dowels (0.31%, 0.62%, and 1.23%) were selected as the main parameters to study and discuss ESS-HPC using direct shear tests. Considering the above four parameters, the direct shear bond stress (DSBS) is discussed. A bond stress–slip curve model of the ESS-HPC and OCS interface was proposed. Combined with the CEB-FIB Model 2010 and the AASHTO Model, the calculation results of the proposed equations are in good agreement with the experimental results within an acceptable range.

## 2. Experimental Program

### 2.1. Material Properties

The OCS was normal concrete that included cement, silica fume, coarse aggregate, fine aggregate, superplasticizer, and water. The mix proportion of the OCS is given in Table 1. The water-to-blinder ratio of the OCS was 0.30. The 28-day compressive strength was 57.39 MPa, measured by the cubic size of 150 mm.

**Figure 1:**
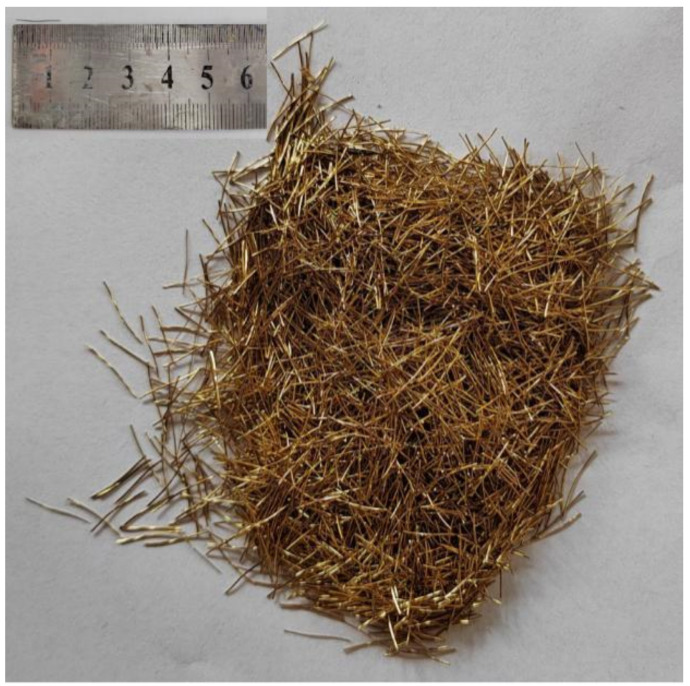
Steel fibers.

The ESS-HPC was a new concrete that included ordinary Portland cement (P.O.52.5), silica fume, fine aggregate (fineness modulus of 2.5~2.7), coarse aggregate (particle size of 5~10 mm), polycarboxylate superplasticizer (water-reducing rate ≥ 30%), straight copper-coated steel fiber (Figure 1), defoaming agent, rubber power, calcium oxide expansive agent, and water. The water-to-binder ratio of the ESS-HPC was 0.32. Steel fibers and an expansive agent were used to improve the ductility and shrinkage behaviors of the ESS-HPC. The volume fraction of the steel fibers was 0.127%. The properties of the straight copper-coated steel fiber and the mixture properties of the ESS-HPC matrix are given in Table 2 and Table 3, respectively. The ESS-HPC showed excellent workability; the mechanical properties are given in Table 4. Four grades of the ESS-HPC’s compressive strength were investigated in this test, in accordance with the GB/T 50081-2019 standard [30]. The 28-day compressive strengths of the specimens with a size of 150 mm × 150 mm × 150 mm were 61.22 MPa (C60), 65.76 Mpa (C65), 71.74 Mpa (C70), and 77.13 MPa (C75). The 7-day and 56-day compressive strengths were 65.43 MPa and 79.38 MPa, respectively. 

### 2.2. Details of the Specimens

**Figure 2:**
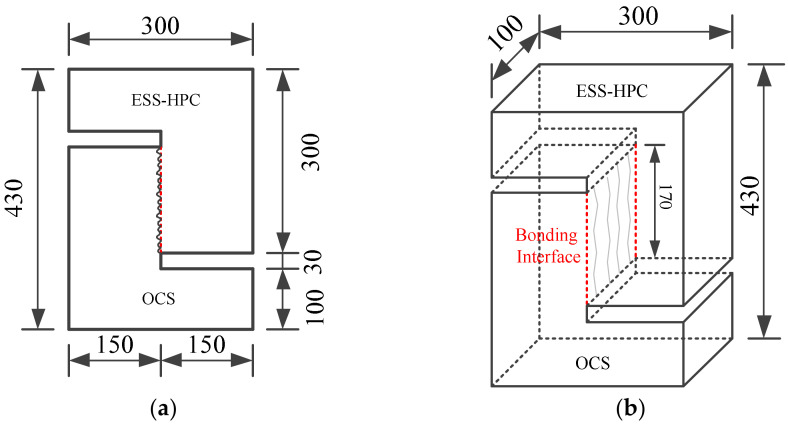
Geometry of direct shear tests (unit: mm). (**a**) Elevation view; (**b**) three-dimensional view.

The Z-type specimens were designed to investigate the shear behavior by direct shear tests. The shear bond stress was evaluated under shear stress conditions and compression and shear loading, respectively [2]. The geometry of the specimens was 100 mm × 300 mm × 430 mm, as shown in Figure 2. The cross-section of the interface was 150 mm × 170 mm.

All specimens were constructed according to the designed geometry. For the specimens without interfacial steel shear dowels, the OCS was firstly carried out in the laboratory according to the GB/T 50081 and GB/T 50080 guidelines [30,31]. The OCS specimens were demolded after one day and cured for another 27 days in a natural environment. Then the specimens were prepared with grooving, chiseling, and drilling to achieve different surface roughness. For the specimens with steel shear dowels, the holes had to be drilled after the OCS surface was treated with a different method. The diameter and depth of the holes were 10 mm and 96 mm, respectively, based on the design requirements. Then the dust was cleaned and the bonding material was injected with the little press. When the bonding material achieved the design strength, the OCS specimens were placed in the corresponding molds again. Simultaneously, the ESS-HPC was added to the molds. Finally, the specimens were cured for one day to demold and another 6 days, 27 days, and 55 days for the failure test.

**Figure 3:**
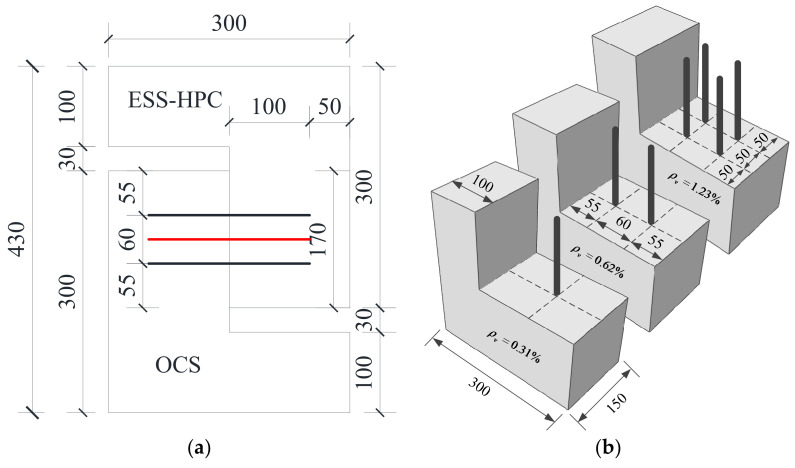
Steel shear dowels (unit: mm). (**a**) Elevation view; (**b**) vertical view.

The geometry of the steel shear dowels is shown in Figure 3. The diameter and embedded length of the hot-rolled ribbed steel shear dowels (*d*) were 8 mm and 96 mm (≥12 *d*), respectively. The elevation view of the steel shear dowels is shown in Figure 3a. The three ratios of the steel shear dowels (*ρ_v_*) at the interface were 0.31%, 0.62%, and 1.23%. The vertical view of the steel shear dowels is shown in Figure 3b. The yield and tensile strength were 540 MPa and 645 MPa, respectively.

Table 5 shows the seventeen groups of Z-type specimens used. Every group included three specimens. Each group was named using a D*i*-*j*-*k*-*l* format. As shown in Table 5, the average value between the ESS-HPC and OCS compressive strength is 64.57 MPa when considering the OCS surface roughness. R2 was established when the ESS-HPC and OCS compressive strength grades and the ESS-HPC curing age were investigated. Except for the ESS-HPC curing age, the 28-day compressive strength was established in other groups. D represents the direct shear test. *i* represents the ESS-HPC curing age, *j* represents the ESS-HPC compressive strength grade, *k* represents the OCS surface roughness degrees, *l* represents the ratio of the steel shear dowels (S1 is 0.31%, S2 is 0.62%, S3 is 1.23%). 70^a^ and 70^b^ represent the 7-day and 56-day compressive strengths. CGM represents the cement-based grouting material that was used to compare with the ESS-HPC. *f_e_* is the ESS-HPC compressive strength. *t* is the ESS-HPC curing age. *τ_c_* and *τ_d_* are the cracking and ultimate shear bond stress provided by the interface (MPa), respectively.

### 2.3. Surface Preparation

**Figure 4:**
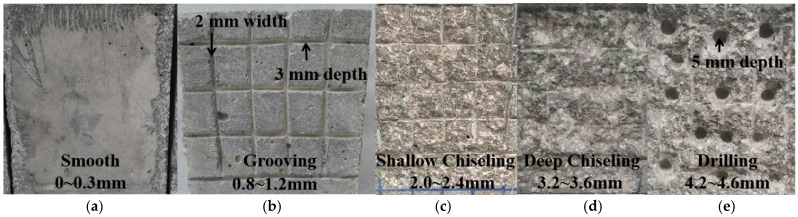
OCS surface roughness in direct shear test. (**a**) R0 (type I); (**b**) R1 (type II); (**c**) R2 (type III); (**d**) R3 (type III); (**e**) R4 (type III).

The sand-filling and coarse aggregate ratio methods were used to evaluate the OCS surface roughness [19]. To investigate the shear bond stress in detail, five types of surface roughness degrees were designed. In this study, it was mainly measured according to the sand-filling method. The surfaces of the OCS specimens were prepared with the grooving, chiseling, or drilling methods. Th original smooth surface without treatment (R0) was selected as the control group, and the average depth was 0.15 mm, as shown in Figure 4a. According to the standard (CECS 242-2016), the type of surface roughness was type I (*n_r_* = H/*δ* < 0.1) [33]. *H* is the average depth via the sand-filling method, and *δ* is the maximum depth of chiseling (10 mm). The coarser surfaces of the OCS were treated with grooving (R1), shallow chiseling (R2), deep chiseling (R3), and drilling (R4). The average depth of R1 was 1.0 mm, and the type of surface roughness was type II (0.1 ≤ *n_r_* ≤ 0.2), as shown in Figure 4b. About 40% to 60% of the coarse aggregate of R2 was visible, and the average depth was 2.2 mm, as shown in Figure 4c. For R3, about 60% to 80% of coarse aggregate was visible, and the average depth was 3.4 mm, as shown in Figure 4d. The average depth of R4 was 4.4 mm, as shown in Figure 3e. The diameter and depth of the drilled holes were 10 mm and 5 mm, respectively. The surface toughness grades of R2, R3, and R4 were type III (*n_r_* > 0.2). In the process of surface preparation, a mesh grid was first sketched on the OCS surface by a marker. Next, the OCS surface was treated with grooving, chiseling, or drilling to different depths. Finally, the coarser surface area of the OCS was cleaned by an air compressor. The five types of surface roughness of the OCS are shown in Figure 4.

### 2.4. Testing Procedure

**Figure 5:**
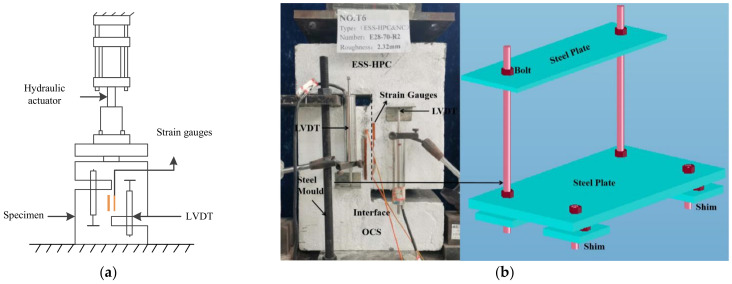
Testing setup. (**a**) Direct shear test; (**b**) steel mold.

Figure 5 demonstrates the setups of the direct shear test. Two linear variable differential transformers (LVDTs) were used to measure the relative slip of the interface, as shown in Figure 5a. Because the specimens of the direct shear test were non-reinforced specimens, a steel mold was used to minimize the premature failure caused by the compression stress concentration and bending moment (see Figure 5b). The specimens were subjected to pure shear loading. The load was applied with an electronic universal testing machine in a displacement-controlled manner. The variations in vertical loading were monitored throughout the entire test process using a load cell connected to a hydraulic jack. The loading rate was carefully controlled to be 0.2 mm/min.

## 3. Experimental Results

### 3.1. Failure Modes

**Figure 6:**
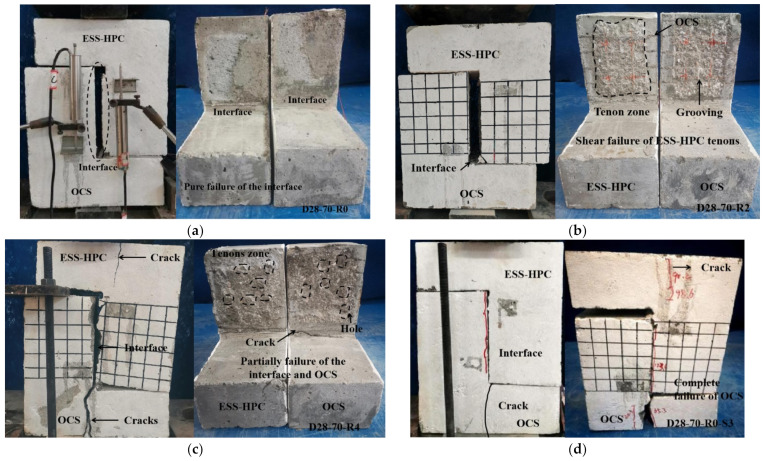

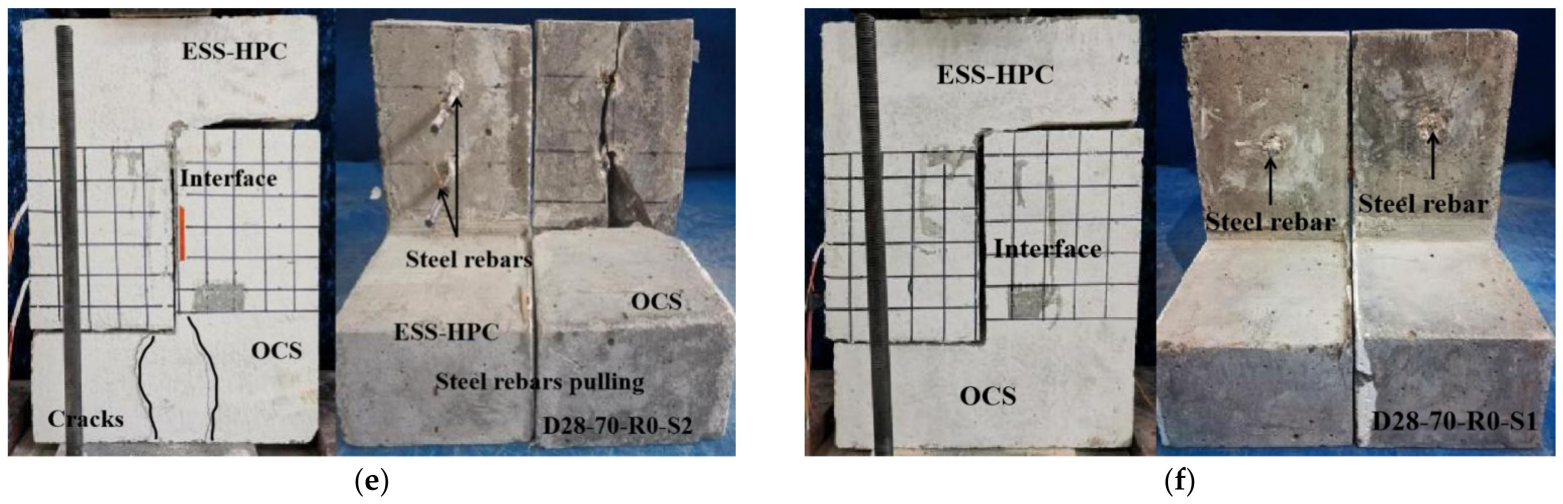
Failure modes of specimen. (**a**) Type A; (**b**) Type A/B; (**c**) Type B; (**d**) Type C; (**e**) Type D; (**f**) Type E.

The failure modes of the Z-type specimens are shown in Figure 6. Six major failure modes are described as follows: (1) Pure failure of the interface (Type A): The pure shear failure occurred along the interface. The ESS-HPC and OCS maintained good integrity after debonding. Moreover, the ratio of the slip plane to the entire failure interface was less than 5%. This type mainly occurred on the OCS with a roughness of R0 and the DCGM28-70-R2 specimen. The specimens showed a brittle failure mode, as shown in Figure 6a. (2) Shear failure of the ESS-HPC tenons (Type A/B): The shear failure was observed vertically along the interface, as shown in Figure 6b. The ESS-HPC tenons in the OCS plane were sheared off. The OCS plane was attached to the thin layer of the ESS-HPC because the surface roughness was treated with chiseling. The shear resistance was provided by the cohesion between the ESS-HPC tenons and OCS. (3) Partial failure of the interface and OCS (Type B): Partial shear failure was observed at the interface and OCS side, as shown in Figure 6c. Cracks were generated along the bottom of the OCS side. Then they propagated to the interface with the increasing load. The interface cohesion failed, and the ESS-HPC tenons in the OCS plane were sheared off. (4) Complete failure of OCS (Type C): The shear failure was observed on the OCS side near the interface. However, the ESS-HPC and OCS were not separated because the cohesion was provided by the steel shear dowels, as shown in Figure 6d. (5) Splitting failure along the steel shear dowels (Type D): This type mainly occurred with ratios of steel shear dowels of 0.62% and 1.23%. The ESS-HPC side showed good integrity and the OCS side was cracked, as shown in Figure 6e. (6) Fracturing of steel shear dowels (Type E): The shear failure mainly occurred with a ratio of steel shear dowels of 0.31%. The steel shear dowels were used to bear the shear load when the load was higher than the cohesion of the interface. The gradual yield of the steel shear dowels and the shear failure are shown in Figure 6f.

**Figure 7:**
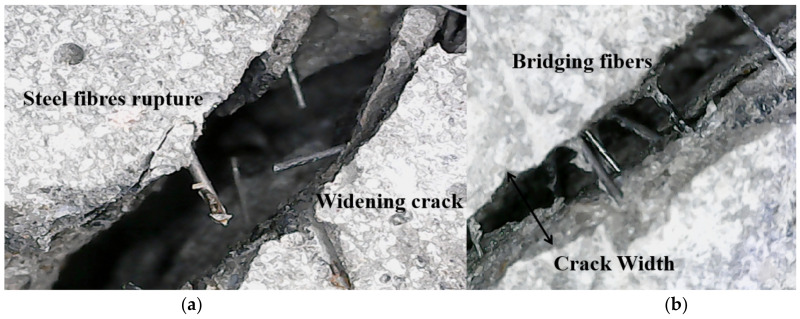
Failure mode and effect of steel fibers. (**a**) Rupture; (**b**) bridging.

**Figure 8:**
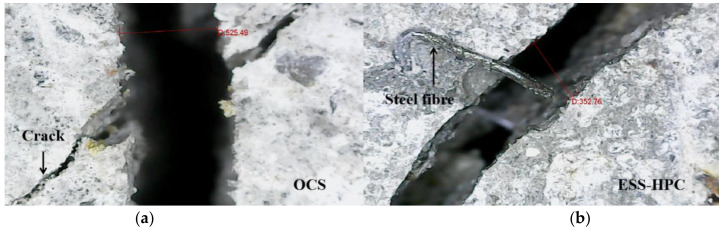
Crack width of the composite. (**a**) OCS; (**b**) ESS-HPC.

Figure 7 shows the failure modes and effects of the steel fibers. Figure 6a demonstrates the steel fiber ruptures on the widening cracks in the failure specimens. The crack-bridging fibers across the narrow crack width can be observed in Figure 6b. The crack width of the composite is shown in Figure 8. The crack width of the OCS was relatively wider than that of the ESS-HPC because the steel fibers in the ESS-HPC hindered crack propagation. It can also be concluded that the micro behavior of the ESS-HPC was better than that of the OCS.

### 3.2. Shear Bond Stress

The DSBS of the Z-type specimens was calculated according to ASTM C882/882M [34], as shown in Equation (1). The values of the shear bond stress are the average values of each group of specimens.

(1) $$\tau_{d}=P_{d}/A_{d}$$

where *P_d_* is the maximum applied load (N), and *A_d_* is the cross-section area of the specimens (mm^2^) and can be taken as a normal value of 2.55 × 10^4^ (mm^2^).

Table 5 shows that the DSBS of the D28-70-R2 specimen is 1.38 times that of the DCGM28-70-R2 specimen. Therefore, the ESS-HPC showed better shear strength behavior than that of the CGM. The effects of the ESS-HPC and OCS compressive strength grade, the ESS-HPC curing age, and the OCS surface roughness on the DSBS were investigated as follows.

#### 3.2.1. Effect of the ESS-HPC and OCS Compressive Strength Grade

**Figure 9:**
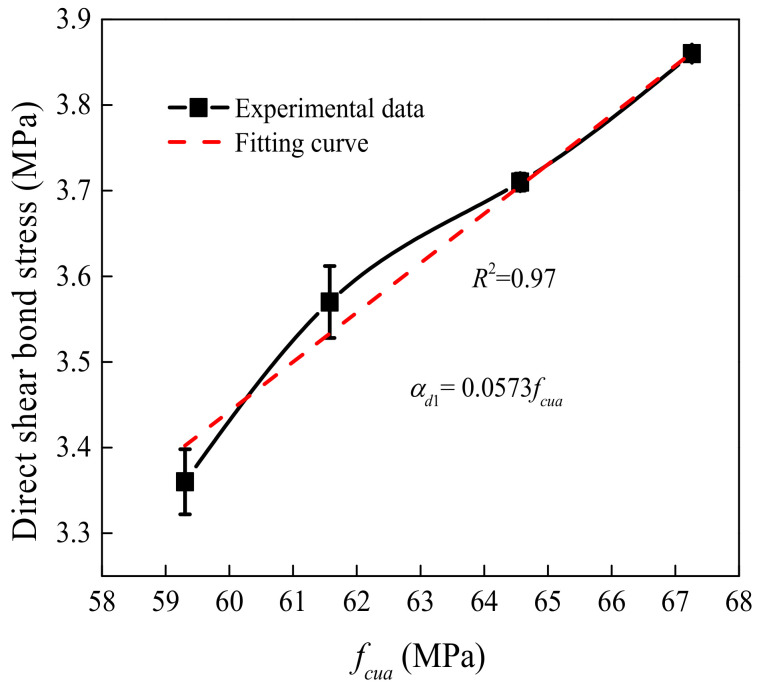
Effect of the ESS-HPC and OCS compressive strength grade on shear bond stress.

Figure 9 displays the effect of the ESS-HPC and OCS compressive strength grade on the shear bond stress. In the figures, *f_cua_* is the average value of the ESS-HPC and OCS compressive strength. α*_d_*_1_ is the bond stress under the influence of the *f_cua_*. The relationship between the DSBS and *f_cua_* was essentially linear. The DSBS of the D28-75-R2 specimen increased by 14.88% compared to the D28-60-R2 specimen. The maximum value of the DSBS increased by 6.25% between two adjacent grades. Therefore, to achieve a higher DSBS, it was necessary to control the concrete grade between the ESS-HPC and OCS.

#### 3.2.2. Effect of the ESS-HPC Curing Age

**Figure 10:**
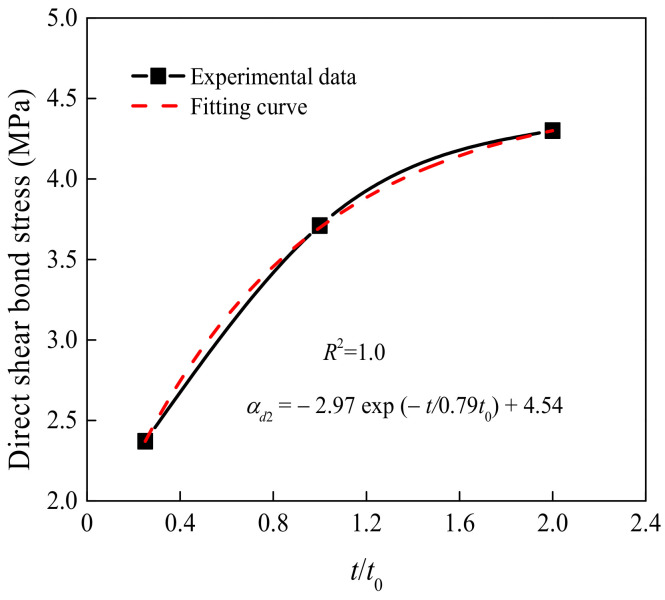
Effect of the ESS-HPC curing age on shear bond stress.

Figure 10 presents the effect of the ESS-HPC curing age on the shear bond stress. In the figures, *t*_0_ is the OCS curing age. α*_d_*_2_ is the bond stress under the influence of *t*/*t*_0._ The relationship between the shear bond stress and the *t*/*t*_0_ is expressed as an exponential function. Because the ESS-HPC strength was lower than that of the OCS at the early curing age, the shear bond stress showed a sharp increasing tendency with the increase in the *t*. The increasing tendency of the shear bond stress became stable when the ESS-HPC strength was higher than that of the OCS. The DSBSs of the D28-70-R2 and D56-70^b^-R2 specimens increased by up to 63.88% and 55.12%, respectively, compared to those of the D7-70^a^-R2 specimen. When the ESS-HPC was selected to strengthen concrete structures, the curing time was more than 56 days. Therefore, the influence of the long-term properties of the interface on the DSBS needs to be further studied. 

#### 3.2.3. Effect of the OCS Surface Roughness

**Figure 11:**
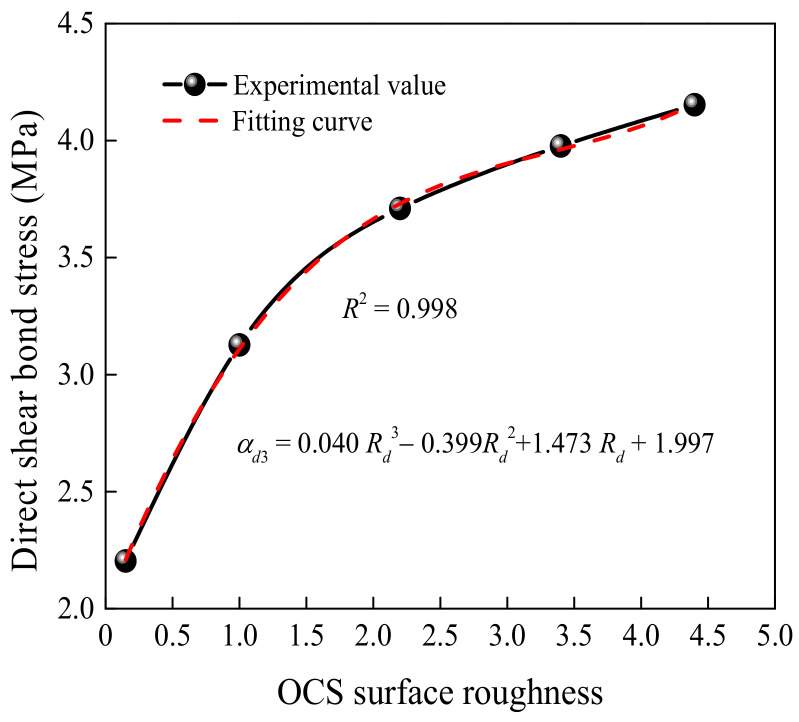
Effect of the OCS surface roughness on shear bond stress.

Figure 11 presents the effect of the OCS surface roughness on the shear bond stress. In the figures, α*_d_*_3_ is the bond stress under the influence of the *R_d_*. The surface roughness values are the average values of each group of specimens. The relationship between the shear bond stress and the OCS surface roughness can be expressed as a cubic function. The OCS surface roughness was treated with grooving and chiseling to achieve the best shear bond strength. This is because a coarser surface can improve the mechanical interlocking. However, the depth of drilling seriously damaged the OCS. Therefore, the surface roughness had a significant influence on the shear bond stress within a certain range. Compared to R0, the DSBSs of the specimens with surface roughness levels of R1, R2, R3, and R4 increased by 42.27%, 68.64%, 80.91%, and 89.09%, respectively. However, the DSBS of the D28-70-R3 specimen merely increased by 7.28% when compared to the D28-70-R2 specimen. Therefore, the optimum OCS surface roughness was R2 when the other parameters were investigated. 

#### 3.2.4. Effect of Ratio of Steel Shear Dowels

**Figure 12:**
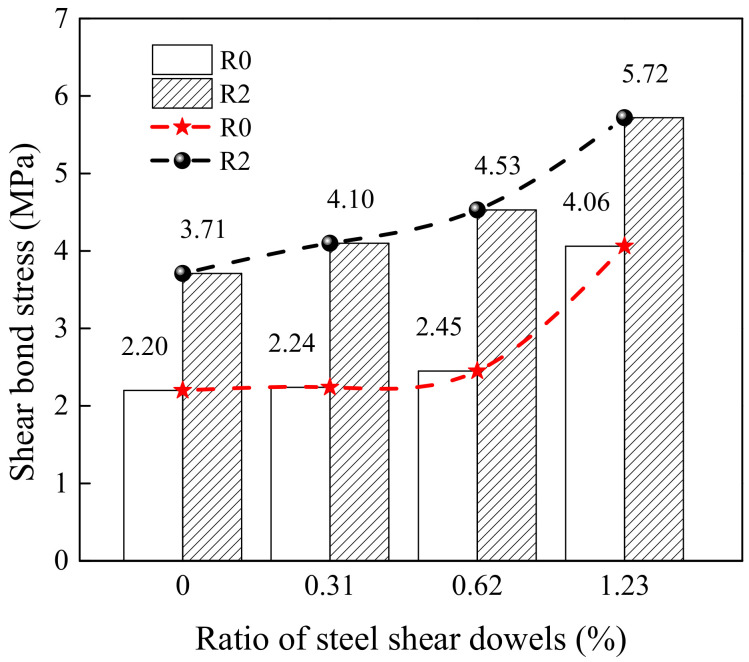
Effect of ratio of steel shear dowels on shear bond stress.

Figure 12 shows the effect of the ratio of steel shear dowels on the DSBS. The R2 surface roughness and steel shear dowels can remarkably improve the shear bond strength. The DSBSs of the D28-70-R0-S3 and D28-70-R2-S3 specimens increased by 84.55% and 54.18%, respectively, when compared to those of the D28-70-R0 and D28-70-R2 specimens. Therefore, the ratios of the steel shear dowels had a significant influence on the DSBS.

#### 3.2.5. Bond Stress–Slip Curves

**Figure 13:**
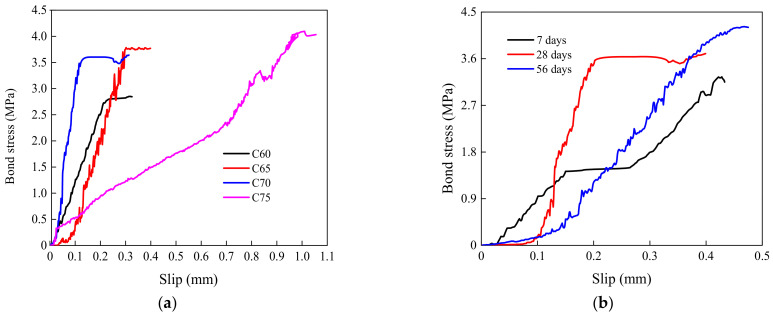

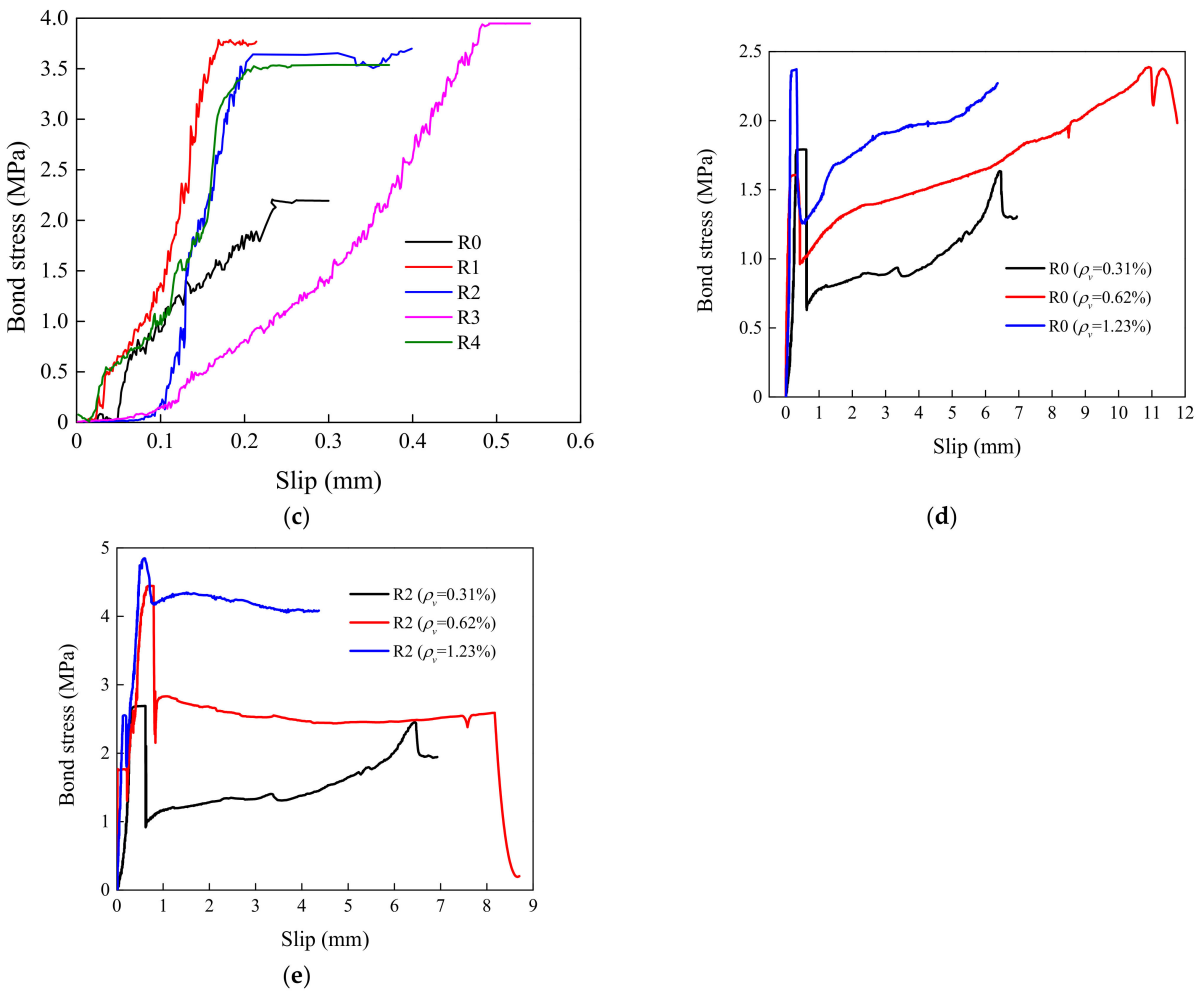
Bond stress–slip curves of the specimens. (**a**) Compressive strength; (**b**) curing age; (**c**) surface roughness; (**d**) specimens with steel shear dowels (R0); (**e**) specimens with steel shear dowels (R2).

The bond stress–slip curves of interfaces have been discussed and analyzed [35,36,37,38]. Figure 13 displays the bond stress–slip curves of the specimens without and with steel shear dowels. The bond stress was calculated by using Equation (1). The interface slips were calculated by the difference between two LVDTs, as shown in Figure 4. As shown in Figure 13a–c, the relationships between the slip and shear bond stress of specimens without steel shear dowels appear to be approximately linear before the ultimate bond stress. The D28-75-R2 specimen achieved a maximum slip equal to 0.99 mm, while that of the other specimens was less than 0.5 mm. There was a stabilization stage in the bond stress–slip curves of all specimens. The length of this stage indicates the influence level of the ESS-HPC and OCS compressive strength grade, the ESS-HPC curing age, and the OCS surface roughness on the bond stress. The order of influence on the bond stress was found to be surface roughness > curing age > compressive strength. It can be concluded that the D28-70-R2 specimen showed the best bond stress resistance.

The bond stress–slip curves of the specimens with steel shear dowels are shown in Figure 13d–e. Because of the steel shear dowels, the bond stress showed a secondary increase and was marginally higher than that of the specimens without steel shear dowels. The maximum slip of the D28-70-R0-S2 specimen was 15.31 mm. Because the interface was valid, the bond stress resistance was mainly provided by the steel shear dowels. The ductility of the specimens with higher steel ratios and coarser surfaces was much better than that of those with lower ratios and smoother surfaces. The bond stresses were not significantly improved when the ratios of steel shear dowels were 0.31% and 0.62%. The shear stress of the specimens with steel shear dowels showed a secondary growth trend, and there was an optimum steel ratio at the interface. When the specimens met the minimum steel ratio, the ratio of the maximum shear stress (*τ_us_*) to the cracking shear stress (*τ_cs_*) was more than 1.0. Using the *τ_us_*/*τ_cs_* and the steel ratio, Equation (2) was obtained. It can be concluded that the relationship between these two parameters was a quadratic function. Deviating from Equation (2), the optimum steel ratio was obtained and the value is 0.83%. Simultaneously, there was a minimum steel ratio to ensure that the shear stress did not significantly decrease after concrete cracking. In this event, the ratio of the maximum shear stress (*τ_us_*) to the cracking shear stress (*τ_cs_*) was 1.0. Therefore, the minimum reinforcement ratio is 0.18%.

(2) $$y=-18817x^{2}+313.58x+0.8523$$

**Figure 14:**
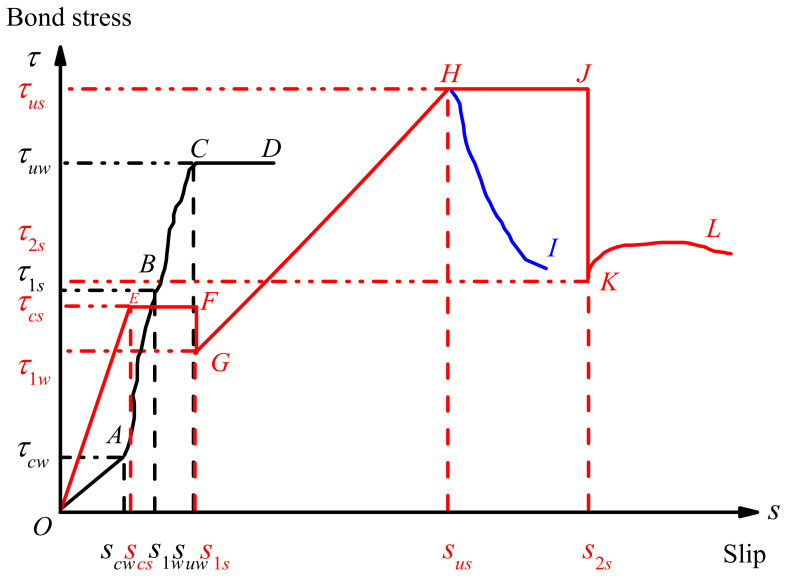
Model of bond stress–slip curves.

The bond stress–slip curves of the specimens without and with steel shear dowels simplified to the model are shown in Figure 14. The black and red in the figure represent the specimens under consideration without and with steel shear dowels, respectively. The model of the specimens without steel shear dowels includes the elastic stage (OA), the elastic–plastic slip stage (AB), the plastic strengthening stage (BC), and the stabilization stage (CD). At the initial loading, the bond stress linearly increased with the increasing slip (OA segment). At the point of A, cracks developed and the corresponding shear stress was defined as the initial cracking bond stress (*τ_cw_*). The bond stress was mainly provided by Vanderwaals, mechanical interlock, and chemical cohesion. Then the specimens entered the elastic–plastic slip stage (AB segment) with the increasing applied load. The relationship between the bond stress and slip can be expressed as a power function, and the bond stress increased more sharply than that of the slip. The bond stress (*τ*_1*s*_) was mainly composed of the chemical adhesion and mechanical interlocking of the aggregates. After the elastic–plastic slip stage, the specimens entered the plastic strengthening stage (BC segment). The bond stress logarithmically increased with the slip at this stage. The increasing rate of bond stress was larger than that of the AB segment. The point of C was the ultimate bond stress (*τ_uw_*). Finally, the specimen entered the stabilization stage (CD segment). The bond stress was stabilized with the increasing amount of slip. Simultaneously, the specimens were broken by types A~B. 

The model of the specimens with steel shear dowels consists of the elastic stage (OE), abrupt transition stage I (EF), descent stage I (FG), plastic strengthening stage (GH), abrupt transition stage II (HJ), descent stage II (JK), and stabilization stage (KL). The curve firstly goes through an elastic stage. However, a higher bond stress than that of the specimens without steel shear dowels was demonstrated. Then the specimens entered the abrupt transition stage I (EF segment). The bond stress (*τ_cs_*) was not improved with the increasing slip at this stage. The steel shear dowels began to provide bond stress when the interfaces failed. During this process, cracks developed and energy dissipation occurred. Therefore, the bond stress decreased from *τ_cs_* to *τ*_1*w*_, and the specimens entered the descent stage I (EF segment). In the plastic strengthening stage, the relationship between the bond stress and slip can be described by a cubic function. The bond stress increased sharply (GH segment), i.e., the bond stress secondarily increased from *τ*_1*w*_ to *τ_us_*. Then the specimens entered the abrupt transition stage II (HJ segment). The steel shear dowels yielded at this stage. The slip increased more than in the abrupt transition stage I. Failure occurred in the descent stage II (JK segment) of the specimens with R0, and the bond stress decreased sharply. However, the bond stress of the OCS with R2 surface roughness specimens decreased to *τ*_2*s*_ (KL segment). The higher ratio of the steel shear dowels showed a stabilization stage (HI segment) even greater than *τ_us_* until broken. Finally, the specimens were broken by types C~E.

## 4. Discussion

Unified expressions of shear bond stress between self-compacting and normal concrete are scarce. Most existing equations have been obtained according to the different sizes, materials, and influence parameters. The influence parameters included the surface roughness, the compressive strength of new and old concrete, the curing age, adhesive strength, etc. Because the ESS-HPC was a new strengthening concrete, the present expression of the shear bond stress was not available. In this study, the shear bond stress was experimentally investigated according to two codes (CEB-FIB Model 2010 [39] and AASHTO Model [40]). The shear bond stress included interlocking, friction, and dowel action. Firstly, the interlocking is discussed. Then the friction coefficient and dowel action are discussed in detail.

### 4.1. CEB-FIB Model 2010

A related investigation proposed the difference in shear bond stress between self-compacting concrete and OCS [41]. However, it was only suitable for the specimen with a depth of surface roughness ranging from 2.0 mm to 2.4 mm. The ratio of steel shear dowels was less than 0.7%, and the compressive strength grade of new concrete changed from C25 to C40. Combined with the experimental results and the method of calculation, α*_di_* (*i* = 1,2,3) was firstly achieved by fitting, as shown in Figure 8, Figure 9 and Figure 10. Then multiplying α*_di_*, the stress was fitted, and *k_d_* was obtained. Finally, the expression of the DSBS could be calculated as follows:

(3) $$\tau_{d}=k_{d}\alpha_{d1}\alpha_{d2}\alpha_{d3}=0.004\left[4.54-2.97\exp\left(-\frac{t}{0.79t_{0}}\right)\right]\left(0.040R_{d}^{3}-0.399R_{d}^{2}+1.473R_{d}+1.997\right)f_{cua}$$

where *τ_d_* is the DSBS (MPa), and *k_d_* is fitted coefficient of the DSBS. 

**Figure 15:**
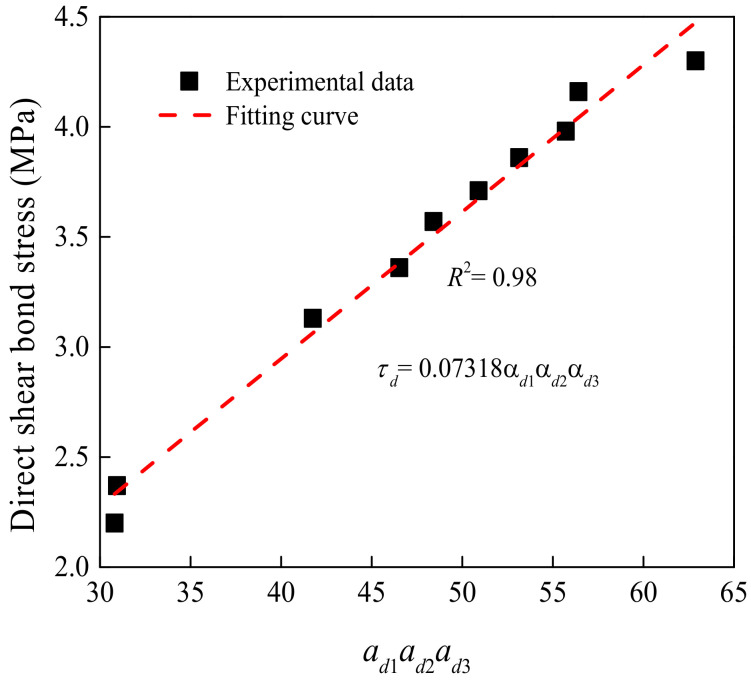
Relationship between shear bond stress and three parameters.

To simplify Equation (3), *k_d_* was obtained by fitting α*_di_* and DSBS. Figure 14 exhibits the relationship between the shear bond stress and three parameters. The DSBS of the interface maintained a linear relationship with the three parameters. As shown in Figure 15, the *k_d_* was 0.07318 and the R square value was 0.98. The average value between the calculated and experimental results is 1.00. The standard deviation (SD) and coefficients of variation (COV) are 0.03. The simplified equation exhibited good computational accuracy.

Taking the practicality and maintenance cycle of the strengthened concrete structure into consideration, the 28-day compressive strength of the ESS-HPC was selected to establish the simplified equation. Finally, the ultimate shear stress of the specimens is shown in Equation (3).

(4) $$\tau_{d}=\left(0.008362R_{d}+0.03256\right)f_{cua}$$

The average value between Equation (4) and the experimental results is 1.00. The SD and COV are 0.02, as given in Table 6. Moreover, the direct shear test was a simple instrumentation and convenient operation and the best method to investigate the shear behavior of the interface between the ESS-HPC and OCS. 

The stress bond stress in the CEB-FIB Model 2010 is measured with the average roughness. The roughness range is similar to that in this investigation. Therefore, Equations (5) and (6) were obtained based on the method of the CEB-FIB Model 2010. Table 7 shows the summary of the test results. Equation (5) shows the shear stress of the specimens without steel shear dowels. It can be concluded that the friction coefficients from the test results (0.02–0.30) are lesser than that of the CEB-FIB Model 2010 (≤0.5, 0.5–1.4). This is because the types of surface roughness in the CEB-FIB Model 2010 were very smooth, smooth, rough, and very rough. The roughened surface was treated with sandblasting or high-pressure water jetting. The OCS surface in this study showed much coarser roughness and was treated with grooving, chiseling, and drilling. Equation (6) shows the shear stress of the specimens with steel shear dowels. It includes the adhesion and friction. The steel shear dowels decreased the friction coefficient and remarkably improved the shear stress. The maximum error between the calculated and experimental results is 3.37%. Therefore, Equations (3)–(6) show good accuracy within an acceptable range.

### 4.2. AASHTO Model

For the specimens with steel shear dowels, the shear bond stress included the summation of the shear contributions from the concrete and steel shear dowels [42]. In this study, the shear contribution from the concrete was calculated using Equation (5). The shear stress expression in the AASHTO Model for cast in situ over precast conditions is as shown in Equation (7).

(7) $$\tau_{A}=0.5+0.6\rho_{v}f_{y}$$

However, the relative error between the calculated and experimental results of the shear stress was 73.83%. Therefore, Equation (7) had to be modified based on the calculation method in the AASHTO Model. Combined with the experimental results, the shear bond stress of the specimens with steel shear dowels could be calculated by Equation (8).

(8) $$\tau_{ds}=\tau_{d}+0.47\rho_{v}f_{y}$$

where *τ_ds_* is the shear bond stress of the specimens with the steel shear dowels (MPa), and *f_y_* is the design tensile strength of the steel shear dowels (MPa).

Taking the related parameters in Equation (8), the average value between the calculated and experimental results is 0.92. The SD and COV are 0.10, as given in Table 8. Therefore, Equation (8) exhibits good computational accuracy of the shear bond stress.

## 5. Conclusions

The direct shear bond stress of the ESS-HPC and OCS interface was investigated. The ESS-HPC and OCS compressive strength grade, the ESS-HPC age, the OCS surface roughness, and the ratio of steel shear dowels were variables. The following main conclusions can be drawn:

1. The failure modes of the specimens in the direct shear test were pure failure of the interface, shear failure of the ESS-HPC tenons, partial failure of the interface and OCS, complete failure of the OCS, splitting failure along the steel shear dowels, and fracturing of the steel shear dowels.

2. The order of influence of the bond stress was found to be surface roughness >curing age > compressive strength. The DSBS improved by 14.88% as the ESS-HPC strength grade changed from C60 to C75. The DSBS of the 28-day and 56-day compressive strength increased by 63.88% and 55.12%, respectively, compared to 7 day compressive strength. The DSBS increased by almost 89.09% for the specimens with R4 surface roughness. 

3. A bond stress–slip model of the interface with and without steel shear dowels was proposed. The model of the specimens without steel shear dowels included the elastic stage, elastic–plastic slip stage, plastic strengthening stage, and stabilization stage. The specimens with steel shear dowels added the abrupt transition stage. To achieve a secondary increase in the shear bond stress, the minimum ratio of steel shear dowels was 0.83%. 

4. The shear bond stress of the interface was established according to the CEB-FIB Model 2010 and AASHTO Models. The calculated results have a good agreement with the experimental results. The excellent shear bond stress can extend the core-filling strengthening method, and the shear behavior of hollow core structures significantly improved.

## Figures and Tables

**Table 1:** Mix proportions of the OCS.

Concrete Grade	Water (kg/m^3^)	Cement (kg/m^3^)	Silica Fume (kg/m^3^)	Coarse Aggregate (kg/m^3^)	Fine Aggregate (kg/m^3^)	Superplasticizer (kg/m^3^)
C55	153	452	50	1077	660	1.0

**Table 2:** Properties of straight copper-coated steel fiber.

Diameter (mm)	Length (mm)	Tensile Strength (GPa)	Density (g/cm^3^)
0.2	13	2.1	7.85

**Table 3:** Mixture properties of the ESS-HPC matrix (kg/m^3^).

Cement	Silica Fume	Fine Aggregate	Coarse Aggregate	Super Plasticizer	Steel Fiber	Defoaming Agent	Rubber Power	Expansive Agent
500	5	600	750	2.5	10	0.1	1.9	50

**Table 4:** Mechanical properties of the ESS-HPC.

Parameter		Results	
Size of coarse aggregate	mm	5–10	
Slump flow	mm	≥610, 0.5 h ≥ 550	
Restrain expansion rate	%	≥0.015	
Setting time [30]	h	Initial	≥1
		Final	≥8
Compressive strength [31]	MPa	3 d	67 ± 3.0
		7 d	74 ± 4.0
		28 d	82 ± 3.0
		60 d	90 ± 2.5
Elastic modulus	GPa	3 d	33 ± 2.0
		28 d	39 ± 1.0
Impermeability grade [32]	P	SF1	
Frost resistance grade	F	≥F200	
Chloride ion penetration	C	≤1000	

**Table 5:** Details of the specimens.

Specimens	*f_e_* (MPa)	*t* (d)	Surface Roughness	Average Depth (mm)	Failure Modes	*τ_c_* (MPa)	*τ_d_* (MPa)
D28-70-R0	71.74	28	Type I	0.15	A	1.88	2.20
D28-70-R1			Type II	1.00	A/B	2.56	3.13
D28-70-R2			Type III	2.20	A/B	2.83	3.71
D28-70-R3			Type III	3.40	B	3.36	3.98
D28-70-R4			Type III	4.40	B	3.47	4.16
D28-60-R2	61.22		Type III	2.20	A/B	2.70	3.36
D28-65-R2	65.76				A/B	3.28	3.57
D28-75-R2	77.13				A/B	3.44	3.86
D7-70^a^-R2	65.43	7			A/B	2.28	2.37
D56-70^b^-R2	79.38	56			B	3.44	4.30
DCGM28-70-R2	/	28			A	2.02	2.68
D28-70-R0-S1	71.74		Type I	0.15	E	2.00	2.24
D28-70-R0-S2					D	1.88	2.45
D28-70-R0-S3					C	2.37	4.06
D28-70-R2-S1			Type III	2.20	D	2.49	4.10
D28-70-R2-S2					C	2.19	4.53
D28-70-R2-S3					C	3.07	5.72

**Table 6:** Comparison of the shear bond stress between the experimental and Equation (4).

Specimens	τ*_exp_* (MPa)	τ*_cal_* (MPa) Equation (4)	τ*_exp_*/*_τcal_*
D28-70-R0	2.20	2.25	1.02
D28-70-R1	3.13	3.05	0.97
D28-70-R2	3.71	3.72	1.00
D28-70-R3	3.98	4.07	1.02
D28-70-R4	4.16	4.12	0.99
D28-60-R2	3.36	3.41	1.02
D28-65-R2	3.57	3.54	0.99
D28-75-R2	3.86	3.87	1.00
Average			1.00
SD			0.02
COV			0.02

**Table 7:** Summary of test results according to the CEB-FIB Model 2010.

Expression of Interface Shear Stress		Specimens	*τ_exp_* (MPa)	Friction Coefficient (*μ*)	*τ_cal_* (MPa)	(*τ_exp_* − *τ_cal_*) × 100% */τ_exp_*
Without steel shear dowels	*τ_d_* = *c* + *μσ* (5)	D28-70-R0	2.20	0.00	2.20	0.00
		D28-70-R1	3.13	0.30	3.10	0.96
		D28-70-R2	3.71	0.50	3.70	0.27
		D28-70-R3	3.98	0.60	4.00	−0.50
		D28-70-R4	4.16	0.70	4.30	−3.37
With steel shear dowels	*τ_ds_* = *c* + *μ*(*σ + ρ_v_f_y_*) (6)	D28-70-R0-S1	2.24	0.02	2.28	−1.81
		D28-70-R0-S2	2.45	0.04	2.40	1.86
		D28-70-R0-S3	4.06	0.25	3.96	2.35
		D28-70-R2-S1	4.02	0.06	3.95	1.71
		D28-70-R2-S2	4.44	0.12	4.32	2.63
		D28-70-R2-S3	5.74	0.30	5.83	−1.53

**Table 8:** Comparison of the shear bond stress between the experimental Equation (8).

Specimens	*τ_exp_* (MPa)	*τ_cal_* (MPa) Equation (8)	*τ_exp_/τ_cal_*
D28-70-R0-S1	2.24	2.68	0.84
D28-70-R0-S2	2.45	3.16	0.77
D28-70-R0-S3	4.06	4.11	0.99
D28-70-R2-S1	4.02	4.19	0.96
D28-70-R2-S2	4.44	4.67	0.95
D28-70-R2-S3	5.74	5.62	1.02
Average			0.92
SD			0.10
COV			0.10

## Data Availability

All data, models, and code generated or used during the study appear in the submitted article.

## Abbreviations

*d*	diameter of steel shear dowels (mm);
*ρ_v_*	ratio of steel shear dowels;
*f_e_*	ESS-HPC compressive strength (MPa);
*R_d_*	surface roughness in direct shear test (mm);
*τ_d_*	direct bond stress (MPa);
*n_r_*	type of surface roughness;
*P_d_*	maximum applied load in direct shear test (N);
*A_d_*	interface area in direct shear test (mm^2^);
*α_d1_*	bond stress under the influence of f_cua_ in direct shear test (MPa);
*f_cua_*	average value of the ESS-HPC and OCS compressive strength (MPa);
*α_d2_*	bond stress under the influence of t/t_0_ in direct shear test (MPa);
*t* and *t_0_*	ESS-HPC and OCS curing age, respectively;
*α_d3_*	bond stress under the influence of R_d_ in direct shear test (MPa);
*τ_ds_*	shear bond stress considering the steel shear dowels (MPa);
*f_y_*	design tensile strength of steel shear dowels (MPa);
*k_d_*	fitting coefficients of the DSBS;
*τ_cw_* and *τ_uw_*	cracking and ultimate bond stress of specimens without steel shear dowels (MPa);
*τ_1s_*	bond stress of specimen without steel shear dowels at the elastic–plastic slip stage (MPa);
*τ_cs_*	bond stress of specimen with steel shear dowels at the elastic stage (MPa);
*τ_1w_*	bond stress of specimen with steel shear dowels at the descent stage I (MPa);
*τ_us_*	ultimate bond stress of specimen with steel shear dowels at the abrupt transition stage II (MPa);
*τ_2s_*	bond stress of specimen with steel shear dowels at the descent stage II (MPa).
